# Identification and validation of hub differential genes in pulmonary sarcoidosis

**DOI:** 10.3389/fimmu.2024.1466029

**Published:** 2024-09-19

**Authors:** Qian Yao, Keting Min, Mengmeng Zhao, Xianqiu Chen, Dong Weng, Ying Zhou

**Affiliations:** ^1^ Department of Respiratory Medicine, Shanghai Pulmonary Hospital, Tongji University School of Medicine, Shanghai, China; ^2^ Clinical Research Center, Shanghai Pulmonary Hospital, Tongji University School of Medicine, Shanghai, China

**Keywords:** pulmonary sarcoidosis, hub genes, protein-protein interaction network, pathway enrichment analysis, immune response

## Abstract

A total of 138 cDEGs were screened from mediastinal lymph nodes and peripheral whole blood. Among them, 6 hub cDEGs including CTSS, CYBB, FPR2, MNDA, TLR1 and TLR8 with elevated degree and betweenness levels were illustrated in protein-protein interaction network. In comparison to healthy controls, CTSS (1.61 vs. 1.05), CYBB (1.68 vs. 1.07), FPR2 (2.77 vs. 0.96), MNDA (2.14 vs. 1.23), TLR1 (1.56 vs. 1.09), and TLR8 (2.14 vs. 0.98) displayed notably elevated expression levels within pulmonary sarcoidosis PBMC samples (P < 0.0001 for FPR2 and P < 0.05 for others), echoing with prior mRNA microarray findings. The most significant functional pathways were immune response, inflammatory response, plasma membrane and extracellular exosome, with 6 hub cDEGs distributing along these pathways. CTSS, CYBB, FPR2, MNDA, TLR1, and TLR8 could be conducive to improving the diagnostic process and understanding the underlying mechanisms of pulmonary sarcoidosis.

## 1 Introduction

Sarcoidosis, a type of multisystem disorder, is marked by non-caseating granulomas formation which can damage potentially all organs (1). Globally, it is estimated that 2-160 individuals per 100,000 suffer from sarcoidosis (2), with an annual occurrence rate of 3-18 per 100,000 (3). Pulmonary sarcoidosis stands as the most frequently researched manifestation, representing the most common type of major organ involvement (1). Worryingly, 10%-20% of pulmonary sarcoidosis patients will progress to fibrotic pulmonary sarcoidosis, leading to a decline in life quality and potentially life-endangering conditions, linked to a death rate ranging from 12% to 18% over a span of 5 years (2, 4).

Diagnosing pulmonary sarcoidosis is difficult due to the lack of specific diagnostic techniques, requiring a thorough grasp of clinical, imaging, and pathological characteristics (5). According to the 2020 guideline from the American Thoracic Society, efforts are crucial to devise easily accessible biological markers, such as those derived from blood, that can serve as economical alternatives to intricate imaging methods and guide therapeutic interventions (1). Typically, histological verification of non-caseating granulomatous inflammation remains essential for a definitive diagnosis (6). Given the intrusive nature and exorbitant expense of tissue biopsy, peripheral blood measurement based on tissue biopsy confirmation holds the potential to aid in uncovering new biomarkers for this disease.

Although the etiology of sarcoidosis remain unclear, genetic factors (7, 8) and environmental or occupational exposures have been reported to be related with the increased risk of the disease (9). The innovative experimental methods of microarrays utilizing extensive gene data could offer fresh perspectives on possible pathogenic processes and related therapies for sarcoidosis (10, 11). Several sarcoidosis genomic studies have been carried out in various bio-samples - including peripheral blood mononuclear cells (PBMC) samples (11), bronchoalveolar lavage (BAL) cells (12) and lung tissues (13, 14). However, there is a lack of study investigating whether the gene expression of PBMC or whole blood, some of which is presumed to traffic to and from diseased tissues, was consistent with the tissue array results (15). The aim of this study was to investigate and validate the key DEGs and mechanistic pathways in biopsy tissues and PBMCs of sarcoidosis patients.

## 2 Materials and methods

### 2.1 Study design and data source

**Figure 1:**
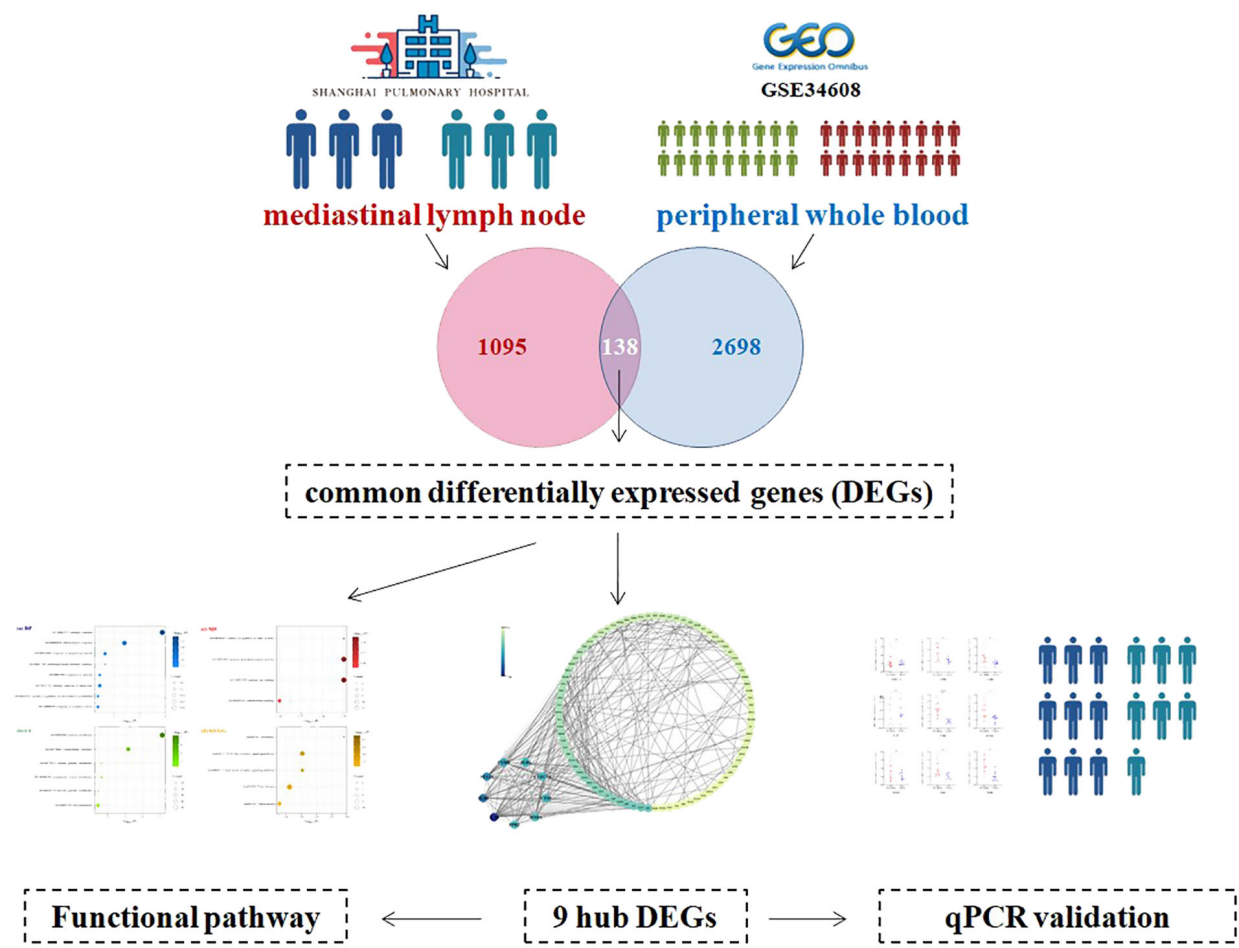
The flowchart of the study.

In Shanghai Pulmonary Hospital, granulomas tissue was obtained from mediastinal lymph nodes in three patients with sarcoidosis (sarcoidosis group) and in three patients with *in situ* lung carcinoma (control group). The mediastinal lymph node granulomas tissues of patients with *in situ* lung carcinoma were pathologically proved to be benign. Then the differentially expressed genes (DEGs) were explored between the two groups. The public transcriptomic dataset GSE34608 was leveraged, in which whole blood gene expression from 18 pulmonary sarcoidosis patients and 18 healthy controls was available. The genes changing in the same direction in two kinds of samples were recognized as common differentially expressed genes (cDEGs). Subsequently, a bioinformatics approach was employed to discern key cDEGs and to investigate the underlying mechanistic pathways. Ultimately, the validation of hub cDEGs expression levels was conducted in PBMC samples from 9 patients with pulmonary sarcoidosis and 7 healthy individuals (**Figure 1**). Sarcoidosis is diagnosed through a combination of rules: a concordant clinical manifestation, histological demonstration of non-necrotizing granuloma formation, and ruling out other potential sources of granulomatous illness (1). The institute ethics committee approved the study protocol (No. k18006).

### 2.2 Hub cDEGs screening

The DEGs from lymph node and whole blood was extracted by R packages “edger” and GEO2R online tools (16), respectively. Genes were classified as DEGs when their absolute values of log_2_ fold change (FC) exceeded 1 and the Benjamini & Hochberg method false discovery rate (FDR) remained below 0.05 (17). The common DEGs (cDEGs) changing in the same direction in two kinds of samples were identified.

The interaction pattern between proteins encoded by cDEGs were retrieved by importing these gained common cDEGs into STRING database (version 9.1), with a combined score over 0.4 as the cut-off value. Following the creation of the protein-protein interaction (PPI) network, the hub cDEGs, proteins with significant biological roles and extensive connections, were pinpointed by assessing their degree and betweenness using CytoHubba plug-ins in Cytoscape 3.8.2 software.

### 2.3 Enrichment analysis

Based on the online Database for Annotation, Visualization, and Integrated Discovery platform, the cDEGs were subjected to Gene Ontology (GO) annotation and Kyoto Encyclopedia of Genes and Genomes (KEGG) pathway. The GO description includes the following 3 domains: biological process (BP), cellular component (CC), and molecular function (MF). KEGG database shows how genes or other molecules act. A stringent threshold of FDR less than 0.05 was employed.

### 2.4 Hub gene verification

Quantitative real-time polymerase chain reaction (qRT-PCR) was employed to validate the expression patterns of top 9 hub cDEGs (degree ≥ 15) in 9 pulmonary sarcoidosis patients diagnosed in Shanghai Pulmonary Hospital and 7 healthy controls that underwent physical examination from September 2023 to December 2023. PBMC total RNA extraction, reverse transcription, and mRNA level quantification was completed in turns by RNAprep pure Cell/Bacteria Kit, FastKing cDNA Kit, and Talent qPCR PreMix, which were purchased from TIANGEN Biotech, China. The sequence of primers used is provided in **Supplementary Table 1**. The mRNA relative expression levels were ascertained using the 2^-ΔΔCt formula, with GAPDH serving as the reference gene for normalization. This research received ethical clearance from the Ethics Committee of Shanghai Pulmonary Hospital (Approval Number: k18006).

### 2.5 Statistical analysis

The characteristics of patients with pulmonary sarcoidosis were described with mean ± standard deviation (SD) or number (%) where appropriate. The relative mRNA expression levels of the verification were compared using Mann-Whitney Test. A two-sided P-value < 0.05 was considered statistically significant in all analysis performed with R software (version 4.3.2).

## 3 Results

### 3.1 Common DEGs

**Figure 2:**
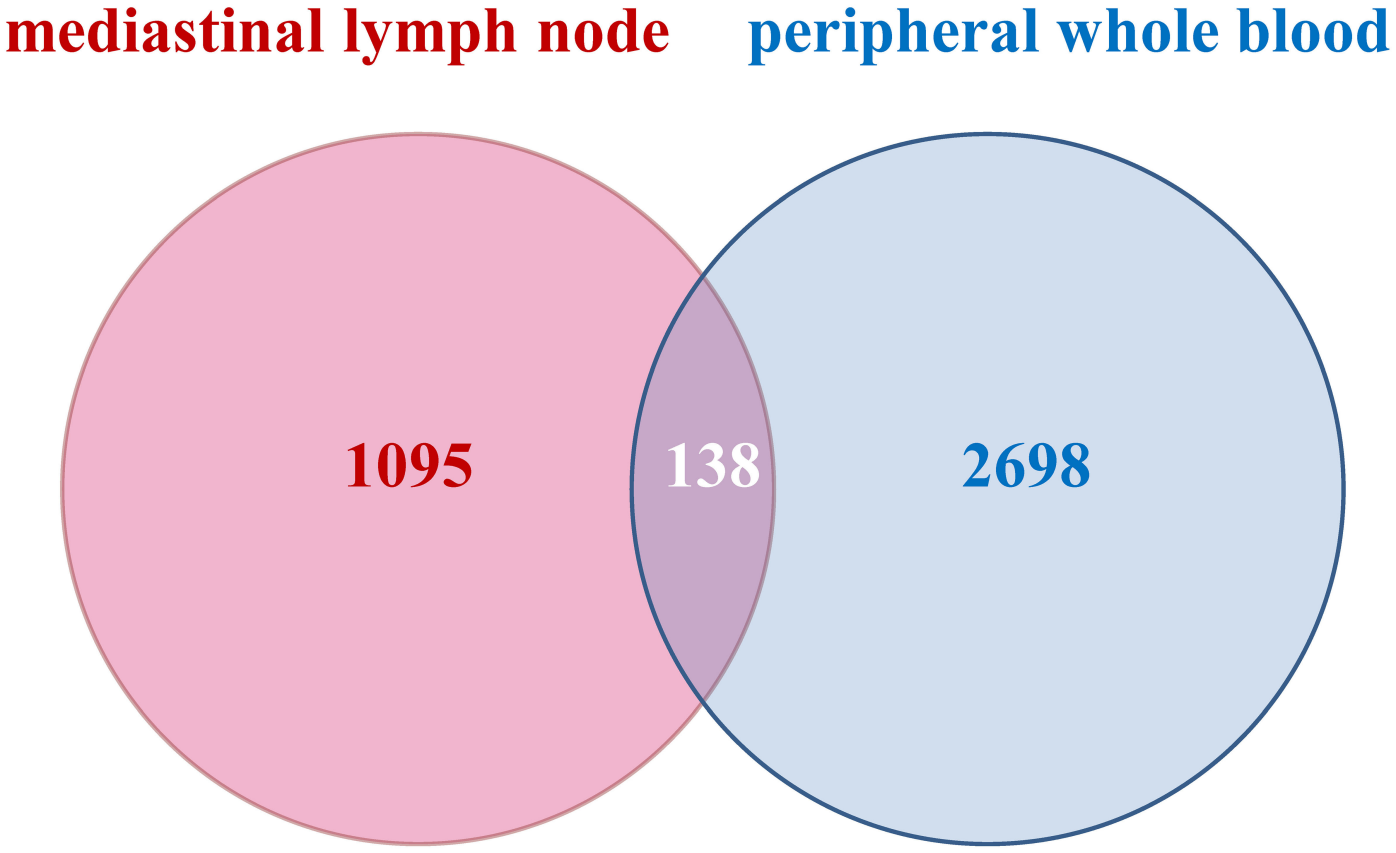
Venn diagrams of common profiles of differentially expressed genes. All identified differentially expressed genes with the same direction from peripheral whole blood and mediastinal lymph node met FDR < 0.05 and |log2 Fold Change|> 1.

To discern the DEGs in pulmonary sarcoidosis compared with normal controls, log_2_FC and FDR computations were employed. There were 2836 and 1233 DEGs in peripheral whole blood and mediastinal lymph node samples, respectively. Finally, 138 cDEGs changing in the same direction were screened from peripheral whole blood and mediastinal lymph nodes samples (**Figure 2**), including 113 genes with augmented expression and 25 genes with diminished expression (**Supplementary Table 2**). As depicted in **Supplementary Figure 1**, these 138 cDEGs effectively differentiated pulmonary sarcoidosis group from the healthy control group, with an observable quantitative predominance of up-regulated genes over down-regulated genes within the sarcoidosis samples.

### 3.2 Hub cDEGs

**Figure 3:**
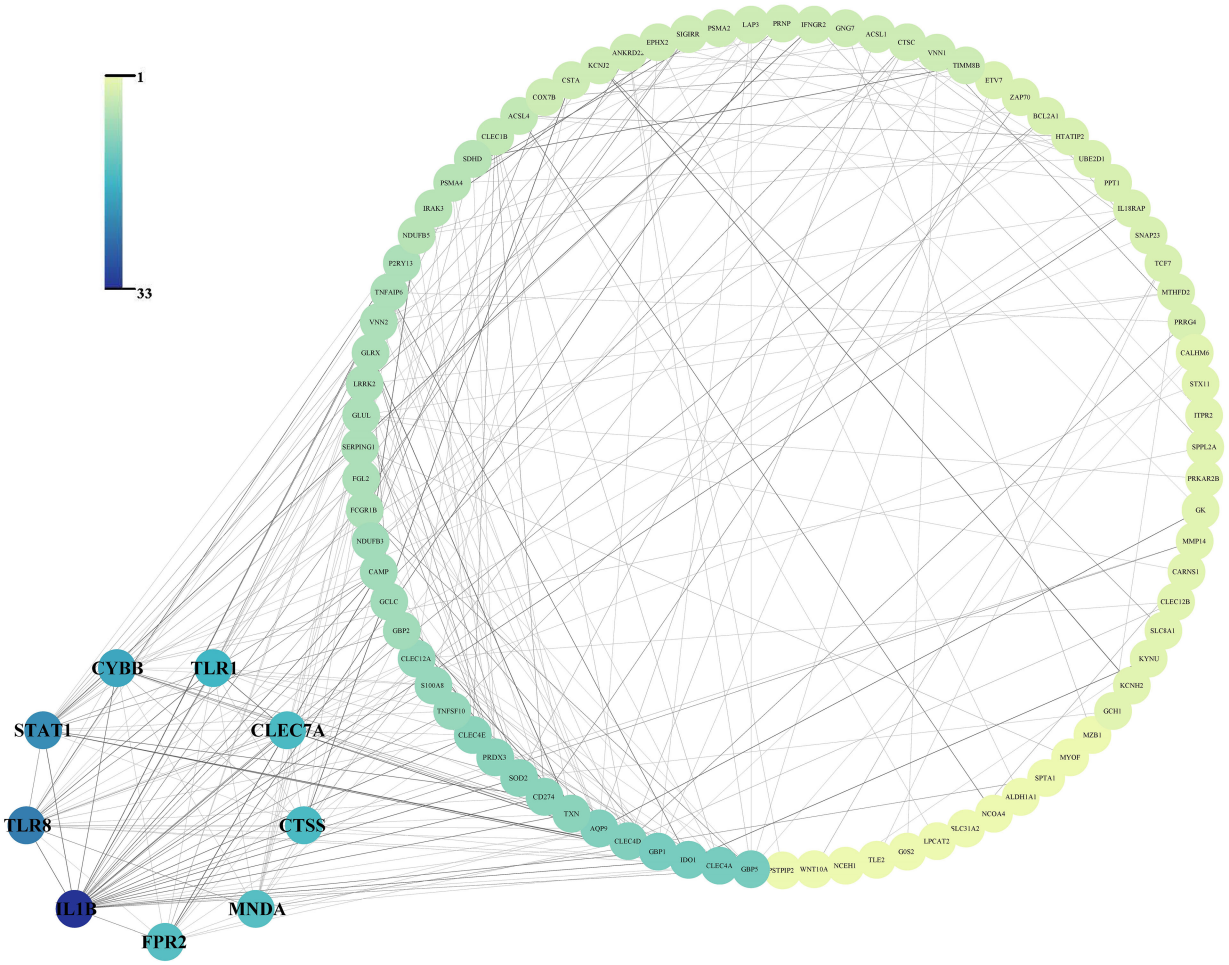
Protein-protein interaction network of the 138 common differentially expressed genes and the 9 hub gene arranged in small circles.

After the submission of 138 cDEGs, 295 PPI pairs were obtained from the STRING database. Within the PPI network, there were 99 nodes symbolizing proteins encoded by the cDEGs, encompassing 86 genes with increased expression, 13 genes with decreased expression, and 295 edges, signifying the linear links among nodes (**Figure 3**).

The PPI network revealed that 9 hub genes, namely IL1B (Gene title: interleukin 1 beta, Degree: 33.0, Betweenness: 2690.87), TLR8 (Gene title: toll like receptor 8, Degree: 24.0, Betweenness: 697.97), STAT1 (Gene title: signal transducer and activator of transcription 1, Degree: 22.0, Betweenness: 1376.07), CYBB (Gene title: cytochrome b-245 beta chain, Degree: 19.0, Betweenness: 622.82), TLR1 (Gene title: toll like receptor 1, Degree: 17.0, Betweenness: 536.26), CLEC7A (Gene title: C-type lectin domain family 7 member A, Degree: 16.0, Betweenness: 152.67), CTSS (Gene title: Cathepsin S, Degree: 16.0, Betweenness: 259.54), FPR2 (Gene title: Formyl peptide receptor 2, Degree: 15.0, Betweenness: 661.97), and MNDA (Gene title: Myeloid cell nuclear differentiation antigen, Degree: 15.0, Betweenness: 325.84) exhibited elevated degree and betweenness levels (**Figure 3**).

### 3.3 Experiment validation

The top 9 cDEGs (degree ≥ 15) expression patterns were further validated by qRT-PCR in 9 pulmonary sarcoidosis patients and 7 healthy controls. The peripheral whole blood samples were isolated to collect PBMC from these participants for the validation. The sarcoidosis group comprised seven women and two men, with a mean age of 52.2 years. No significant disparity was noted in terms of age and gender among patients with pulmonary sarcoidosis when compared to the healthy control group.

**Figure 4:**
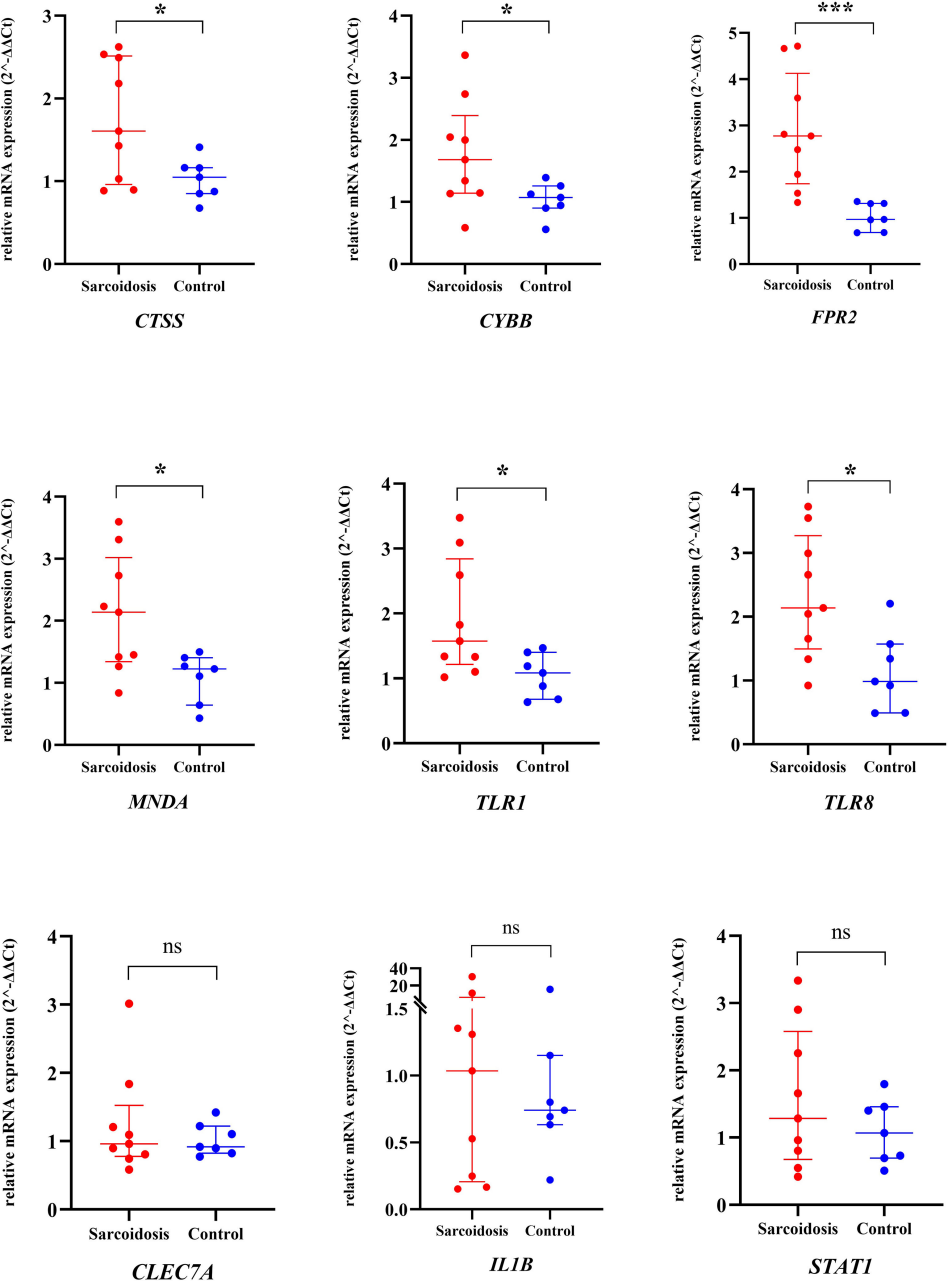
The relative mRNA expression of 9 hub genes in PBMC samples from pulmonary sarcoidosis patients and healthy controls using qRT-PCR. The long line and short line indicated the median value and interquantile range. The two groups were compared using an unpaired two-sided Mann-Whitney Test. *P<0.05; ***P<0.0001; ns, non-significant; qRT-PCR, quantitative real-time polymerase chain reaction; PBMC, peripheral blood mononuclear cells.

In comparison to healthy controls, CTSS (1.61 vs. 1.05), CYBB (1.68 vs. 1.07), FPR2 (2.77 vs. 0.96), MNDA (2.14 vs. 1.23), TLR1 (1.56 vs. 1.09), and TLR8 (2.14 vs. 0.98) displayed notably elevated median expression levels within pulmonary sarcoidosis PBMC samples (*P* < 0.0001 for FPR2 and *P* < 0.05 for others) (**Figure 4**), echoing with prior mRNA microarray findings in both peripheral blood and mediastinal lymph node specimens. Nevertheless, CLEC7A (0.96 vs. 0.92), IL1B (1.04 vs. 0.74), and STAT1 (1.28 vs. 1.07) demonstrated a tendency towards increased expression in pulmonary sarcoidosis patients, these alterations did not reach statistical significance.

### 3.4 Functional pathway

**Figure 5:**
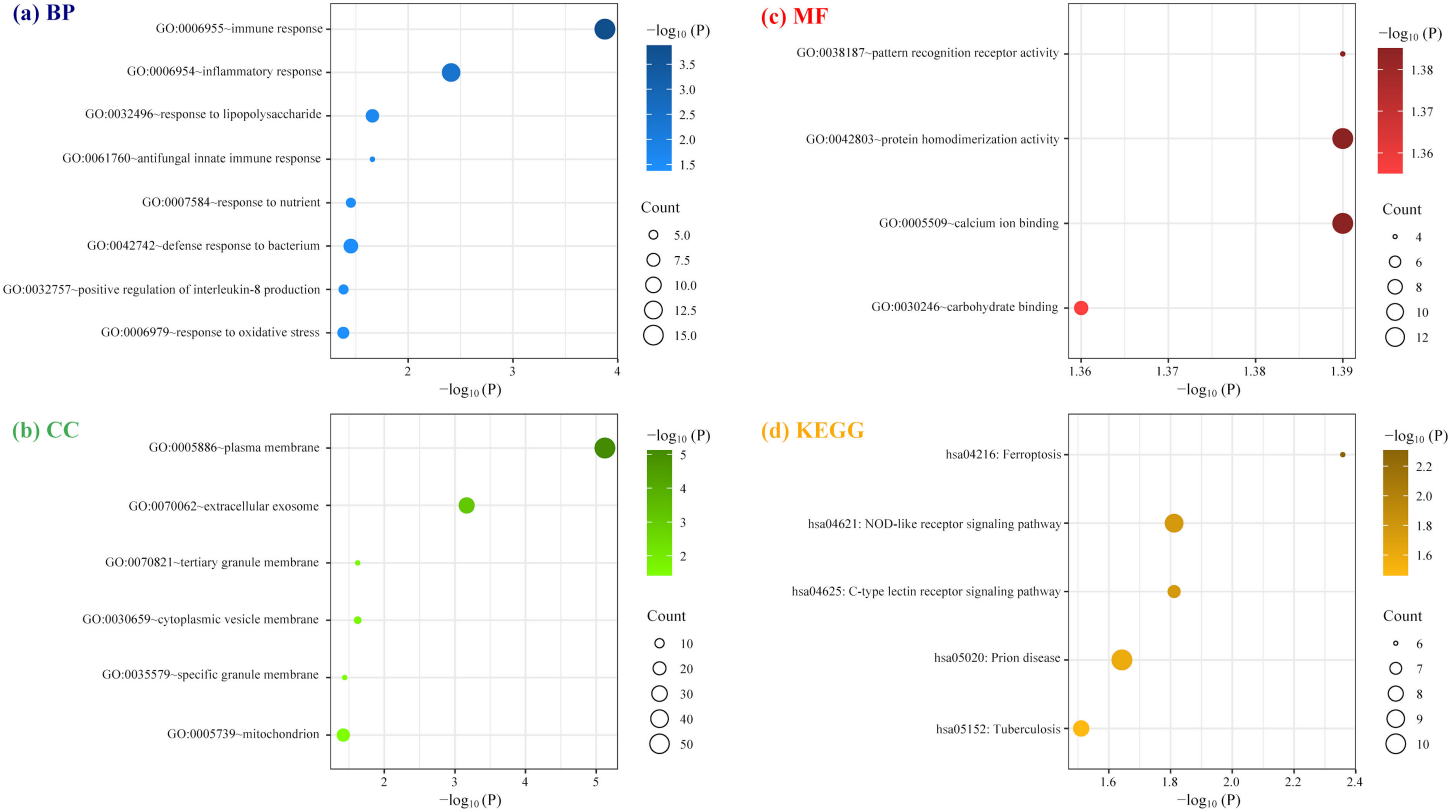
Enrichment analysis of cDEGs involved in the protein-protein interaction network constructed by STRING database. **(A)** Biological Process (BP); **(B)** cellular component (CC); **(C)** molecular function (MF); **(D)** Kyoto Encyclopedia of Genes and Genomes (KEGG).

As illustrated in **Figure 5** (**Supplementary Table 3**), GO and KEGG analyses for the 138 cDEGs showed that these genes displayed prominent enrichment (FDR < 0.05) in eighteen GO terms: eight BP terms, six CC terms and four MF terms, alongside five KEGG pathways. Among them, the most significant pathway (FDR < 0.01) were GO:0006955~immune response (*P* = 1.33E-07; count number: 15, hub gene: CTSS and TLR1), GO:0006954~inflammatory response (P = 7.79E-06; count number: 12, hub gene: TLR1, CYBB, TLR8, and FPR2), GO:0005886~plasma membrane (*P* = 3.56E-08; count number: 51, hub gene: CYBB, TLR1, TLR8, and FPR2), and GO:0070062~extracellular exosome (*P* = 6.04E-04; count number: 27, hub gene: MNDA).

## 4 Discussion

In this study, 138 cDEGs between patients with pulmonary sarcoidosis and healthy controls were screened from both mediastinal lymph nodes and peripheral whole blood. Among them, 6 hub cDEGs including CTSS, CYBB, FPR2, MNDA, TLR1, and TLR8 were identified by PPI network and subsequently verified by qRT-PCR using PBMC samples in the newly recruited validation population. GO and KEGG analyses for one hundred and thirty-eight cDEGs identified the most significant functional pathways were immune response, inflammatory response, plasma membrane and extracellular exosome, with 6 hub genes distributing along these pathways.

This study indicated that TLR1 and CTSS were enriched in immune response pathway, and TLR1, CYBB, TLR8, and FPR2 were enriched in inflammatory response pathway. Sarcoidosis is typified by the presence of epithelioid granulomas, with its initial pathogenesis likely rooted in the innate immune response. This involves phagocytic cells employing pattern recognition receptors in an attempt to eliminate substances that are either sparingly or entirely insoluble (18). Toll like receptors (TLRs), including TLR1 and TLR8, are a kind of membrane-bound pattern recognition receptors, responsible for identifying various pathogen-associated molecular patterns, which may also explain why TLR1 and TLR8 were enriched in plasma membrane pathway. TLR1 and TLR8 recognize cell wall components of mycobacteria (19) and single-stranded viral RNA, respectively (20). Although the relationship of these two receptors with pulmonary sarcoidosis has not been extensively explored, they have been reported to be linked to cutaneous sarcoidosis (21) and cardiac sarcoidosis (22). In addition, our GO MF results showed that TLR8 was enriched for pattern recognition receptor activity, suggesting that the alteration of TLR8 activity may be involved in the pathogenesis of sarcoidosis. CTSS is responsible for coding Cathepsin S, a lysosomal enzyme playing a crucial role in the breakdown of the invariant or Ii chain, which in turn ensures the inhibition of antigen loading onto the major histocompatibility complex class II molecules (23). Thus, an overproduction of Cathepsin S might lead to early breakdown of Ii, sporadic accumulation of major histocompatibility complex class II, and the consequent initiation of an autoimmune reaction. Tanaka et al. quantified serum level cathepsin S for eighty nine healthy donors and one hundred and seven sarcoidosis patients using enzyme-linked immunosorbent test, revealing that CTSS may serve as a novel serum indicator for sarcoidosis, which was derived from transcriptomic profiling of alveolar macrophages (24). The protein product of CYBB often participates in defense against microbial pathogens by generating reactive oxygen species (25). Werner et al. discovered that a link between CYBB deficiency and impaired bacterial clearance, along with heightened granuloma development in the lung using a *Cybb* ^−/−^ mice model (26). Christophi et al. observed an increase in CYBB mRNA in tissue biopsy specimens from sarcoidosis-specific granulomas, which is consistent with our results (27). Besides exogenous triggers, FPR2 could bind self-compounds such as serum amyloid A (SAA), crucial in the progression of sarcoidosis (28). MNDA regulates the pathogen-induced type I interferon cascade in human monocytes, essential for anti-viral reactions, and also instigates autoimmunity in cases of dysregulation (29). Our result found that MNDA was enriched in extracellular exosome pathway, and it is well known that virus species frequently interact with exosomes (30). A multitude of immunological debates suggest that the insufficient removal of viral particles, along with diverse immunodeficiencies, could play a role in sarcoidosis disease (31). Thus, our study supports previous research showing sarcoidosis as an immune-validated disease with a complex disease pathogenesis (32), underscoring the significance of alterations in receptor genes in its development.

There are several limitations in our research. Firstly, the restricted quantity of clinical specimens utilized necessitates validation of our findings within a more extensive sarcoidosis patient group. Secondly, our confirmation process was restricted to the expression patterns of DEGs in PBMC samples, without exploring their mechanistic roles in animal models. Thirdly, while IL1B exhibits the maximum degree and betweenness in the PPI network, its validation in PBMCs did not yield statistically robust results, possibly due to the inherent instability of this gene expression in PBMC. Consequently, additional investigations are warranted in subsequent research. Fourthly, since it is ethically impossible to obtain mediastinal lymph node tissue from healthy people, this study used pathologically benign mediastinal lymph node granulomas tissue from patients with *in situ* lung carcinoma instead.

## 5 Conclusions

In conclusion, the current study identified 138 cDEGs among patients with pulmonary sarcoidosis samples in both mediastinal lymph node granulomas and peripheral whole blood. CTSS, CYBB, FPR2, MNDA, TLR1, and TLR8 could be conducive to improving the diagnostic process and understanding the underlying mechanisms of pulmonary sarcoidosis, and were further verified in PBMC samples using qRT-PCR. The most significant functional pathway was immune response, inflammatory response, plasma membrane and extracellular exosome, with 6 hub genes distributing along these pathways. These revelations offer novel perspectives on the etiology of pulmonary sarcoidosis, thereby establishing a premise for the development of targeted therapeutic approaches.

## Data Availability

The datasets presented in this study can be found in online repositories. The names of the repository/repositories and accession number(s) can be found in the article/**Supplementary Material.**
