# Supplementary material for: Identification and validation of hub differential genes in pulmonary sarcoidosis
Source: Front Immunol. 2024 Sep 19;15:1466029. doi: 10.3389/fimmu.2024.1466029 (PMC11446782; doi:10.3389/fimmu.2024.1466029)
Supplement: Supplementary file 1 [file DataSheet1.doc]

Supplementary Material

# Supplementary Tables

## Supplementary Table 1. The sequence of primers of hub genes

| **Gene** | **NCBI GeneID** | **Gene title** | **Sequence (5' -> 3')** | |
| --- | --- | --- | --- | --- |
| GAPDH | 2597 | glyceraldehyde-3-phosphate dehydrogenase | Forward Primer | GGAGTCCACTGGCGTCTTCA |
|  |  |  | Reverse Primer | GTCATGAGTCCTTCCACGATACC |
| CYBB | 1536 | cytochrome b-245 beta chain | Forward Primer | CTCACTGGCTGGGATGAGTC |
|  |  |  | Reverse Primer | ACTCAGGGTTTCAGCCAAGG |
| STAT1 | 6772 | signal transducer and activator of transcription 1 | Forward Primer | CCTGCTCCCTCTCTGGAATG |
|  |  |  | Reverse Primer | TCAACCGCATGGAAGTCAGG |
| IL1B | 3553 | interleukin 1 beta | Forward Primer | CCTCCAGGGACAGGATATGGA |
|  |  |  | Reverse Primer | ACGCAGGACAGGTACAGATT |
| TLR1 | 7096 | toll like receptor 1 | Forward Primer | AGCTGACAGAGCAAGCAAAGA |
| Supplementary Table 1 (Continued). The sequence of primers of hub genes | | | | |
| **Gene** | **NCBI GeneID** | **Gene title** | **Sequence (5' -> 3')** | |
|  |  |  | Reverse Primer | GAACTGCGACCCGAAGGTAT |
| TLR8 | 51311 | toll like receptor 8 | Forward Primer | GGCCATCATCGACAACCTCA |
|  |  |  | Reverse Primer | AGCTCTTACAGATCCGCTGC |
| FPR2 | [2358](https://www.ncbi.nlm.nih.gov/gene/?term=2358) | formyl peptide receptor 2 | Forward Primer | ACTAATGACACGGCTGCCAA |
|  |  |  | Reverse Primer | GCTGGAACTGGCATTAGGGT |
| CLEC7A | 388512 | C-type lectin domain family 7 member A | Forward Primer | GGGCTCTCAAGAACAATGGAA |
|  |  |  | Reverse Primer | TCTGAAACAACAGCTATCCTGGT |
| MNDA | 4332 | myeloid cell nuclear differentiation antigen | Forward Primer | CGCAAGCTGAAACTGGTGTG |
|  |  |  | Reverse Primer | AGCGGAAGTTGTTTGTTGCATT |
| CTSS | 1520 | cathepsin S | Forward Primer | GCCCAGTGTCTGTTGGTGTA |
|  |  |  | Reverse Primer | TCACCATAGCCAACCACAAGT |

## Supplementary Table 2. The statistical metrics for 138 common differentially expressed genes

| **Gene** | **peripheral whole blood** | | **mediastinal lymph node** | |
| --- | --- | --- | --- | --- |
|  | **log_2_Fold Change** | **adj.P** | **log_2_Fold Change** | **adj.P** |
| GBP1 | 2.5024 | 1.38E-12 | 2.4928 | 2.31E-16 |
| SNX10 | 2.3303 | 2.54E-14 | 3.3713 | 5.13E-23 |
| CSTA | 2.1714 | 1.28E-11 | 1.7749 | 1.80E-02 |
| GBP5 | 2.1049 | 2.46E-09 | 2.1298 | 4.00E-13 |
| COX7B | 2.0486 | 6.18E-10 | 1.3503 | 1.09E-02 |
| LRRK2 | 1.9847 | 3.06E-13 | 1.3671 | 3.55E-05 |
| ANKRD22 | 1.9831 | 2.75E-07 | 4.7742 | 3.60E-37 |
| CLEC1B | 1.9583 | 6.56E-12 | 2.4196 | 2.65E-02 |
| CLEC4D | 1.9368 | 5.90E-09 | 4.9347 | 3.25E-13 |
| MMP14 | 1.9133 | 1.31E-12 | 2.4815 | 2.37E-10 |
| CD274 | 1.8876 | 9.93E-11 | 1.8847 | 2.64E-10 |
| BCL2A1 | 1.8818 | 2.80E-12 | 1.5955 | 4.67E-07 |
| MAP3K7CL | 1.8584 | 9.11E-10 | 2.1432 | 9.83E-05 |
| STAT1 | 1.7717 | 1.19E-11 | 2.0460 | 6.01E-13 |
| PSMA4 | 1.7519 | 3.77E-11 | 1.0883 | 5.02E-03 |
| TXN | 1.7505 | 4.45E-14 | 2.2056 | 2.34E-10 |
| TLR8 | 1.7489 | 6.81E-15 | 3.1347 | 1.15E-22 |

## Supplementary Table 2 (Continued). The statistical metrics for 138 common differentially expressed genes

| **Gene** | **peripheral whole blood** | | **mediastinal lymph node** | |
| --- | --- | --- | --- | --- |
|  | **log_2_Fold Change** | **adj.P** | **log_2_Fold Change** | **adj.P** |
| CLEC4E | 1.7324 | 8.88E-12 | 3.1313 | 5.56E-11 |
| NBN | 1.7273 | 9.49E-14 | 1.0004 | 8.54E-03 |
| GLRX | 1.7265 | 2.76E-10 | 1.3400 | 2.25E-05 |
| FGL2 | 1.7086 | 2.53E-14 | 1.4713 | 1.95E-06 |
| FPR2 | 1.7002 | 1.63E-12 | 3.3045 | 5.19E-08 |
| ACSL4 | 1.7001 | 1.65E-12 | 1.4062 | 1.21E-05 |
| PRKAR2B | 1.6949 | 2.53E-09 | 1.9897 | 1.26E-07 |
| CPVL | 1.6769 | 5.32E-12 | 1.1017 | 2.60E-03 |
| SOD2 | 1.6731 | 1.61E-10 | 1.9070 | 7.50E-08 |
| FCGR1B | 1.6731 | 2.96E-07 | 2.3511 | 1.83E-03 |
| LPCAT2 | 1.6277 | 8.38E-14 | 1.8486 | 5.88E-07 |
| CLEC7A | 1.6055 | 8.57E-12 | 1.7143 | 1.91E-05 |
| NDUFB3 | 1.5989 | 3.65E-10 | 1.7970 | 7.75E-05 |
| MCTP1 | 1.5726 | 1.42E-10 | 1.1755 | 7.75E-03 |
| CLEC4A | 1.5581 | 2.23E-13 | 1.7470 | 1.43E-04 |
| VNN2 | 1.5506 | 2.41E-09 | 1.6959 | 1.38E-08 |
| DYNLT1 | 1.5436 | 1.70E-13 | 1.0520 | 2.29E-03 |

## Supplementary Table 2 (Continued). The statistical metrics for 138 common differentially expressed genes

| **Gene** | **peripheral whole blood** | | **mediastinal lymph node** | |
| --- | --- | --- | --- | --- |
|  | **log_2_Fold Change** | **adj.P** | **log_2_Fold Change** | **adj.P** |
| VNN1 | 1.5391 | 2.28E-09 | 1.8101 | 1.36E-03 |
| G0S2 | 1.5324 | 4.71E-06 | 1.5187 | 5.71E-03 |
| DPYD | 1.5219 | 1.77E-16 | 1.1163 | 7.39E-04 |
| QPCT | 1.4921 | 3.27E-12 | 1.9334 | 1.68E-05 |
| PRDX3 | 1.4855 | 1.17E-10 | 1.2743 | 6.60E-04 |
| GK | 1.4748 | 4.37E-11 | 1.3023 | 7.27E-03 |
| TNFAIP6 | 1.4706 | 3.94E-06 | 5.0539 | 5.08E-20 |
| ARHGAP18 | 1.4690 | 2.90E-12 | 1.1106 | 4.08E-03 |
| FAM198B | 1.4656 | 1.16E-08 | 1.5300 | 1.45E-05 |
| IL1B | 1.4607 | 3.43E-12 | 1.5737 | 2.05E-02 |
| NCOA4 | 1.4582 | 2.36E-12 | 1.4172 | 1.73E-05 |
| P2RY13 | 1.4566 | 1.10E-12 | 1.6520 | 5.61E-07 |
| KCNJ2 | 1.4510 | 1.51E-08 | 2.0064 | 6.55E-08 |
| IDO1 | 1.4397 | 4.00E-05 | 2.7518 | 8.67E-07 |
| UBE2D1 | 1.4357 | 1.09E-11 | 1.9500 | 1.12E-07 |
| TLR1 | 1.4283 | 9.08E-15 | 1.0380 | 8.92E-04 |
| KYNU | 1.4227 | 2.53E-10 | 1.7452 | 1.25E-06 |

## Supplementary Table 2 (Continued). The statistical metrics for 138 common differentially expressed genes

| **Gene** | **peripheral whole blood** | | **mediastinal lymph node** | |
| --- | --- | --- | --- | --- |
|  | **log_2_Fold Change** | **adj.P** | **log_2_Fold Change** | **adj.P** |
| S100A8 | 1.4159 | 3.86E-09 | 4.4144 | 2.11E-13 |
| ETV7 | 1.4073 | 3.62E-04 | 1.5564 | 1.67E-02 |
| NDUFB5 | 1.4016 | 1.48E-10 | 1.1997 | 6.65E-04 |
| MTHFD2 | 1.3901 | 2.01E-10 | 1.3495 | 5.04E-05 |
| LINC01094 | 1.3897 | 2.40E-10 | 1.9760 | 6.35E-04 |
| ALDH1A1 | 1.3777 | 1.64E-10 | 1.3029 | 1.04E-02 |
| CLEC12A | 1.3733 | 2.69E-05 | 3.2158 | 6.80E-09 |
| FAM26F | 1.3689 | 2.02E-05 | 1.9889 | 1.59E-06 |
| TFEC | 1.3501 | 1.29E-12 | 1.5267 | 2.62E-04 |
| ZDHHC20 | 1.3438 | 1.84E-08 | 1.0871 | 1.03E-03 |
| DOCK4 | 1.3422 | 2.37E-10 | 1.1958 | 1.58E-02 |
| ITGBL1 | 1.3394 | 2.27E-05 | 1.8973 | 2.03E-02 |
| STX11 | 1.3341 | 1.81E-12 | 1.5503 | 3.21E-07 |
| CPD | 1.3333 | 3.77E-12 | 1.0693 | 2.25E-03 |
| TNFSF10 | 1.3301 | 3.51E-09 | 1.0438 | 1.88E-03 |
| FAM96A | 1.3037 | 1.29E-09 | 1.3073 | 6.64E-04 |
| ARRDC4 | 1.3034 | 4.55E-08 | 1.5503 | 5.74E-05 |

## Supplementary Table 2 (Continued). The statistical metrics for 138 common differentially expressed genes

| **Gene** | **peripheral whole blood** | | **mediastinal lymph node** | |
| --- | --- | --- | --- | --- |
|  | **log_2_Fold Change** | **adj.P** | **log_2_Fold Change** | **adj.P** |
| LACTB | 1.3032 | 1.24E-13 | 1.4358 | 1.18E-04 |
| CAMP | 1.2726 | 6.66E-04 | 3.8693 | 1.59E-06 |
| SPTA1 | 1.2721 | 8.41E-09 | 2.3339 | 3.11E-02 |
| ATP6AP2 | 1.2605 | 2.41E-12 | 1.1649 | 1.91E-04 |
| GLUL | 1.2598 | 6.68E-12 | 1.2892 | 5.54E-03 |
| C5orf15 | 1.2471 | 3.25E-09 | 1.0565 | 2.40E-03 |
| MNDA | 1.2293 | 9.77E-11 | 1.7156 | 2.94E-05 |
| PRRG4 | 1.2133 | 3.09E-06 | 1.8328 | 1.35E-04 |
| TIMM8B | 1.2076 | 3.00E-07 | 1.1021 | 2.26E-02 |
| CLEC12B | 1.2034 | 3.10E-04 | 1.9393 | 2.93E-02 |
| GCH1 | 1.1972 | 1.13E-10 | 1.8259 | 2.64E-08 |
| PRNP | 1.1866 | 1.86E-09 | 1.1403 | 3.20E-03 |
| LAP3 | 1.1851 | 5.51E-07 | 1.4675 | 5.73E-07 |
| SNAP23 | 1.1850 | 2.49E-10 | 1.0930 | 4.17E-04 |
| NCEH1 | 1.1774 | 1.25E-09 | 1.0558 | 9.52E-04 |
| ACSL1 | 1.1760 | 5.12E-09 | 1.2540 | 2.80E-02 |
| GBP2 | 1.1665 | 4.12E-08 | 1.2215 | 1.76E-04 |

## Supplementary Table 2 (Continued). The statistical metrics for 138 common differentially expressed genes

| **Gene** | **peripheral whole blood** | | **mediastinal lymph node** | |
| --- | --- | --- | --- | --- |
|  | **log_2_Fold Change** | **adj.P** | **log_2_Fold Change** | **adj.P** |
| ITPR2 | 1.1573 | 9.76E-12 | 1.0201 | 1.26E-03 |
| DDX3Y | 1.1547 | 6.63E-06 | 7.8010 | 6.41E-04 |
| IRAK3 | 1.1544 | 3.04E-11 | 1.8143 | 1.18E-08 |
| ARL6IP5 | 1.1530 | 1.91E-09 | 1.0236 | 1.04E-02 |
| AQP9 | 1.1447 | 7.89E-09 | 2.3183 | 5.94E-03 |
| PLBD1 | 1.1216 | 2.06E-08 | 1.3154 | 9.80E-03 |
| OSGIN2 | 1.1164 | 7.31E-12 | 1.1010 | 6.48E-03 |
| IL18RAP | 1.1050 | 6.22E-04 | 1.0443 | 3.23E-02 |
| PSTPIP2 | 1.1015 | 1.18E-11 | 2.5277 | 1.62E-16 |
| CYBB | 1.0990 | 3.92E-07 | 2.2524 | 3.03E-14 |
| PSMA2 | 1.0910 | 1.04E-08 | 1.1179 | 7.99E-03 |
| GALNT1 | 1.0908 | 3.94E-09 | 1.2100 | 3.99E-03 |
| SERPING1 | 1.0902 | 8.51E-04 | 1.5218 | 1.53E-05 |
| GCLC | 1.0855 | 3.31E-06 | 1.0203 | 4.98E-03 |
| PPT1 | 1.0795 | 2.09E-07 | 1.6464 | 4.86E-08 |
| EPB41L3 | 1.0735 | 1.48E-07 | 1.0150 | 6.18E-03 |
| SLC31A2 | 1.0709 | 9.78E-07 | 1.3325 | 2.20E-03 |

## Supplementary Table 2 (Continued). The statistical metrics for 138 common differentially expressed genes

| **Gene** | **peripheral whole blood** | | **mediastinal lymph node** | |
| --- | --- | --- | --- | --- |
|  | **log_2_Fold Change** | **adj.P** | **log_2_Fold Change** | **adj.P** |
| PLXDC2 | 1.0684 | 2.92E-10 | 1.5119 | 1.17E-02 |
| UGP2 | 1.0652 | 1.82E-12 | 1.1586 | 8.14E-04 |
| MYOF | 1.0648 | 7.55E-06 | 2.1348 | 1.70E-07 |
| SPPL2A | 1.0481 | 2.29E-12 | 1.2173 | 1.21E-04 |
| HTATIP2 | 1.0441 | 4.19E-13 | 1.1178 | 1.72E-03 |
| SLC8A1 | 1.0435 | 2.05E-09 | 1.0934 | 7.67E-04 |
| IFNGR2 | 1.0300 | 1.23E-10 | 1.2764 | 7.85E-05 |
| CTSS | 1.0264 | 1.12E-10 | 1.6241 | 1.53E-05 |
| CREG1 | 1.0189 | 3.12E-07 | 1.1005 | 1.56E-02 |
| CTSC | 1.0020 | 4.69E-08 | 1.8160 | 2.60E-10 |
| SDHD | 1.0015 | 5.59E-10 | 1.1882 | 3.64E-04 |
| KLHL34 | -1.0499 | 4.42E-11 | -1.8579 | 3.48E-02 |
| GNG7 | -1.0935 | 9.34E-15 | -1.3591 | 2.93E-02 |
| VWA7 | -1.1529 | 4.94E-04 | -1.6270 | 2.88E-02 |
| MZB1 | -1.1778 | 6.56E-04 | -2.0668 | 9.71E-05 |
| CARNS1 | -1.1847 | 6.95E-15 | -1.3379 | 3.47E-02 |
| KCNH2 | -1.2087 | 3.11E-14 | -1.8864 | 1.92E-02 |

## Supplementary Table 2 (Continued). The statistical metrics for 138 common differentially expressed genes

| **Gene** | **peripheral whole blood** | | **mediastinal lymph node** | |
| --- | --- | --- | --- | --- |
|  | **log_2_Fold Change** | **adj.P** | **log_2_Fold Change** | **adj.P** |
| BHLHA15 | -1.2245 | 1.12E-02 | -2.3337 | 4.20E-02 |
| CDH24 | -1.2595 | 5.24E-07 | -1.2245 | 2.61E-02 |
| DOCK3 | -1.2898 | 1.68E-07 | -1.7197 | 9.86E-03 |
| LTBP4 | -1.3023 | 1.40E-15 | -1.4547 | 6.31E-05 |
| CACNA1I | -1.3709 | 1.18E-16 | -1.7590 | 2.55E-04 |
| LTK | -1.3808 | 5.86E-09 | -2.5315 | 6.52E-07 |
| EPHX2 | -1.3826 | 8.19E-06 | -1.2636 | 2.45E-02 |
| SMG1P5 | -1.3889 | 1.35E-15 | -1.4318 | 6.60E-04 |
| BAIAP3 | -1.3910 | 3.23E-09 | -1.3607 | 1.56E-02 |
| WNT10A | -1.3920 | 1.55E-17 | -1.4896 | 9.44E-04 |
| PAQR6 | -1.7464 | 1.50E-10 | -1.3086 | 4.31E-02 |
| SIGIRR | -1.7671 | 3.94E-13 | -1.0358 | 8.21E-03 |
| TLE2 | -2.0020 | 5.58E-16 | -1.1313 | 3.66E-02 |
| LMF1 | -2.0543 | 9.99E-12 | -1.4000 | 3.10E-02 |
| KIAA1683 | -2.0914 | 9.12E-14 | -1.9456 | 1.06E-03 |
| GRASP | -2.2495 | 2.18E-16 | -1.4578 | 2.33E-02 |
| PGGHG | -2.3978 | 6.53E-14 | -1.4404 | 6.20E-06 |

## Supplementary Table 2 (Continued). The statistical metrics for 138 common differentially expressed genes

| **Gene** | **peripheral whole blood** | | **mediastinal lymph node** | |
| --- | --- | --- | --- | --- |
|  | **log_2_Fold Change** | **adj.P** | **log_2_Fold Change** | **adj.P** |
| ZAP70 | -2.4472 | 3.27E-14 | -1.0489 | 7.59E-03 |
| TCF7 | -2.7728 | 3.68E-13 | -1.4716 | 3.83E-04 |

## Supplementary Table 3. Enrichment analysis of cDEGs involved in the protein-protein interaction network constructed by STRING database.

| **Category** | **Term** | **Count** | **FDR** |
| --- | --- | --- | --- |
| MF | GO:0038187~pattern recognition receptor activity | 4 | 4.05E-02 |
| MF | GO:0042803~protein homodimerization activity | 13 | 4.05E-02 |
| MF | GO:0005509~calcium ion binding | 13 | 4.05E-02 |
| MF | GO:0030246~carbohydrate binding | 7 | 4.31E-02 |
| CC | GO:0005886~plasma membrane | 51 | 7.05E-06 |
| CC | GO:0070062~extracellular exosome | 27 | 6.40E-04 |
| CC | GO:0070821~tertiary granule membrane | 5 | 2.24E-02 |
| CC | GO:0030659~cytoplasmic vesicle membrane | 6 | 2.24E-02 |
| CC | GO:0035579~specific granule membrane | 5 | 3.42E-02 |
| CC | GO:0005739~mitochondrion | 17 | 3.58E-02 |
| BP | GO:0006955~immune response | 15 | 1.31E-04 |
| BP | GO:0006954~inflammatory response | 12 | 3.85E-03 |
| BP | GO:0032496~response to lipopolysaccharide | 7 | 2.17E-02 |
| BP | GO:0061760~antifungal innate immune response | 4 | 2.17E-02 |
| BP | GO:0007584~response to nutrient | 5 | 3.48E-02 |
| BP | GO:0042742~defense response to bacterium | 8 | 3.48E-02 |
| BP | GO:0032757~positive regulation of interleukin-8 production | 5 | 4.09E-02 |
| BP | GO:0006979~response to oxidative stress | 6 | 4.11E-02 |
| KEGG | hsa04216:Ferroptosis | 6 | 4.25E-03 |
| KEGG | hsa04621:NOD-like receptor signaling pathway | 9 | 1.50E-02 |
| KEGG | hsa04625:C-type lectin receptor signaling pathway | 7 | 1.50E-02 |
| KEGG | hsa05020:Prion disease | 10 | 2.21E-02 |
| KEGG | hsa05152:Tuberculosis | 8 | 2.99E-02 |

MF, molecular function; CC, cellular component; BP, Biological Process; KEGG, Kyoto Encyclopedia of Genes and Genomes

# Supplementary Figures


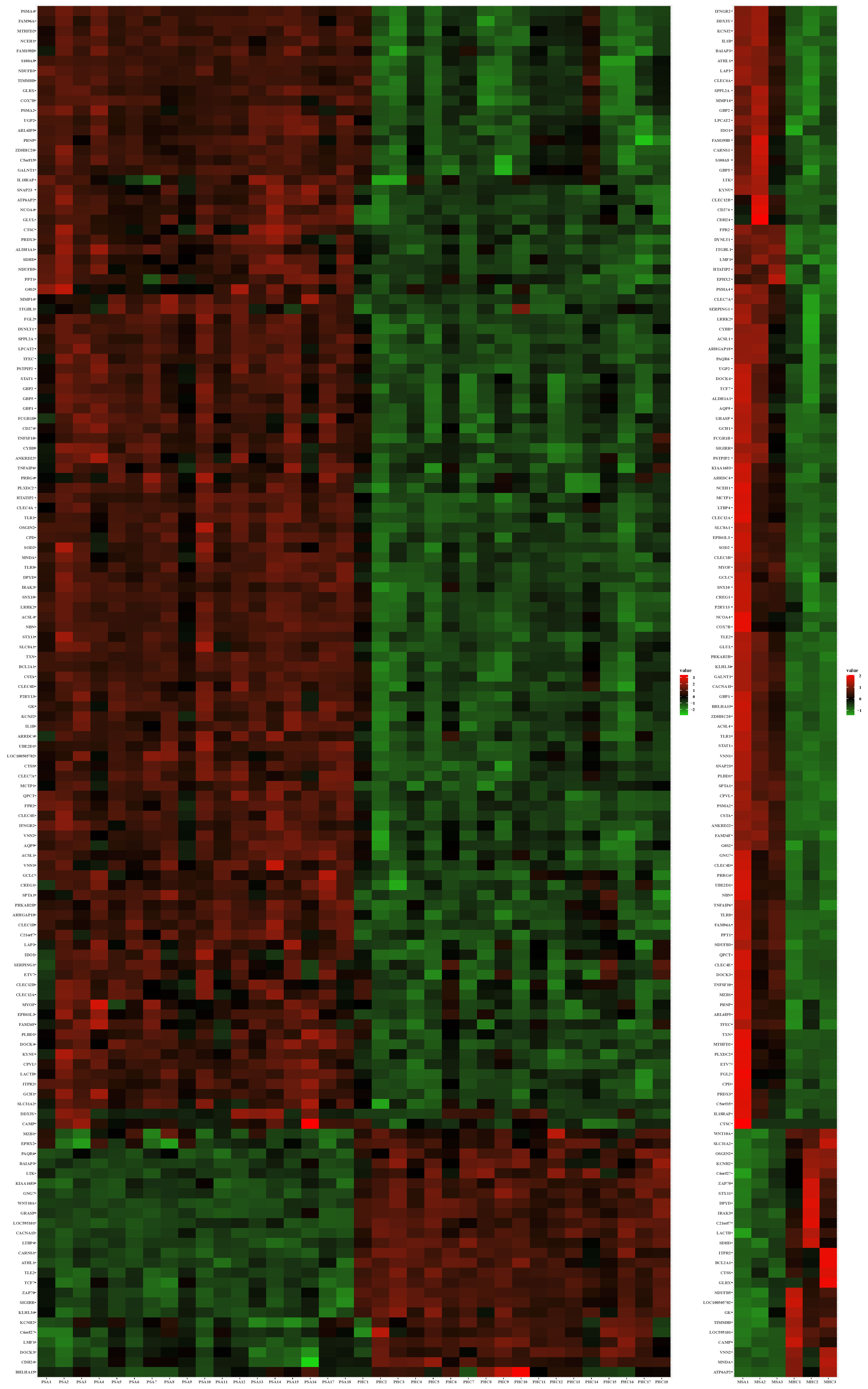


**Supplementary Figure 1.** Heatmap of 138 common differentially expressed genes. The color red and green indicate higher expression and lower expression, respectively. Abbreviations: PSA, peripheral whole blood sample in pulmonary sarcoidosis group; PHC, peripheral whole blood sample in healthy control group; MSA, mediastinal lymph nodes in pulmonary sarcoidosis group; MHC, mediastinal lymph nodes sample in normal control group.
